# The effects of ice vest pre-cooling on skin blood flow at rest and during exercise in the heat

**DOI:** 10.1186/2046-7648-4-S1-A127

**Published:** 2015-09-14

**Authors:** Mike Price, Matthew Maley

**Affiliations:** 1 Department of Applied Science and Health, Coventry University, Coventry, CV1 5FB, UK; 2 Extreme Environments Laboratory, Department of Sport and Exercise Science, University of Portsmouth, Portsmouth, UK

## Introduction

Ice vest pre-cooling has been show to lower rectal temperature during intermittent exercise in hot conditions but only after 40 min of exercise [1]. The authors suggested that the ice vest may have initiated a strong local cutaneous vasoconstrictor response reducing skin blood flow [2] and thus the cooling potential, until increases in body temperature and skin blood flow occurred later in exercise. Therefore, the purpose of this study was to determine whether ice vest pre-cooling reduces skin blood flow during intermittent exercise in the heat compared to a no cooling control.

## Methods

Eight male participants volunteered to take part in the study. Following preliminary tests for peak oxygen uptake and peak power output on a cycle ergometer participants undertook either ice vest pre-cooling in cool conditions (mean (SD) air temperature 19.7 (0.4) °C) for 20 min (PRE; using Arctic Heat cooling vests) or a no cooling control (CON) prior to 5 min seated rest in the heat and 45 min of intermittent cycling in the heat (mean (SD) air temperature 35.4 (0.4) °C, 26.3 (4.1) % RH). Participants undertook two further trials involving no exercise to determine the reliability of the cooling procedure. Rectal (T_re_) and aural (T_au_) temperature, mean skin temperature (T_ms_; [3]), skin blood flow (SkBF; Laser Doppler at the bicep, chest, back and thigh) and ratings of perceived thermal strain (RPTS) were recorded throughout the trial. Data were analysed using two-way analysis of variance with repeated measures on both factors (trial × time) using SPSS v17.0.

## Results

Back skin temperature was cooler following PRE (20.3 (5.0) °C) than for CON (30.3 (1.6) °C; P < 0.05) but demonstrated greater intra and inter-individual variation during PRE (~5.0°) when compared to CON (~1.6°C). T_au _and T_re _increased by similar amounts during exercise for both PRE and CON (T_au _~1.2 °C, T_re _~0.6 °C; P > 0.05). However, T_au _was cooler from 5 to 25 min of exercise during PRE (P < 0.05) whereas T_re _was cooler only at 45 min of exercise during PRE. T_ms _and RPTS were significantly lower during the pre-cooling period (27.1 (2.6) °C; 2.6 (0.8) °C) compared to CON (30.9 (1.0) °C; 3.6 (0.8) °C, respectively; P < 0.05) but did not differ during exercise in the heat. RPTS values were consistent between and within participants. Although no trial × time interactions were observed for SkBF sites a main effect for trial for the back site indicated a decrease in SkBF during the pre-cooling period (-40 (26) %) when compared to CON (9 (45) %; P < 0.05).

## Discussion

Ice vest pre-cooling resulted in decreased T_au _and T_re_, but at different time points, during intermittent cycle exercise in the heat. Back skin temperature responses to cooling were varied and likely explained by the closeness of fit of commercially available ice vests with subsequent effects on local skin blood flow.

## Conclusion

The delay in reducing T_re _following ice vest pre-cooling may be a result of deep body temperature measurement site rather than changes in local skin blood flow per se.
